# Intrathoracic versus Cervical Anastomosis after Resection of Esophageal Cancer: A matched pair analysis of 72 patients in a single center study

**DOI:** 10.1186/1477-7819-10-159

**Published:** 2012-08-06

**Authors:** Christian D Klink, Marcel Binnebösel, Jens Otto, Gabriele Boehm, Klaus T von Trotha, Ralf-Dieter Hilgers, Joachim Conze, Ulf P Neumann, Marc Jansen

**Affiliations:** 1 Department of General, Visceral and Transplantation Surgery, Aachen, Germany; 2 Department of Medical Statistics, RWTH Aachen University Hospital, Aachen, Germany; 3 Department of General, Visceral and Minimal Invasive Surgery, Helios Hospital Emil von Behring, Berlin, Germany; 4 Department of General, Visceral and Transplantation Surgery, RWTH Aachen University Hospital, Pauwelsstr. 30, 52074, Aachen, Germany

**Keywords:** Esophageal cancer, Esophageal resection, Cervical anastomosis, Intrathoracic anastomosis, Transthoracic, Transhiatal

## Abstract

**Background:**

The aim of this study was to analyze the early postoperative outcome of esophageal cancer treated by subtotal esophageal resection, gastric interposition and either intrathoracic or cervical anastomosis in a single center study.

**Methods:**

72 patients who received either a cervical or intrathoracic anastomosis after esophageal resection for esophageal cancer were matched by age and tumor stage. Collected data from these patients were analyzed retrospectively regarding morbidity and mortality rates.

**Results:**

Anastomotic leakage rate was significantly lower in the intrathoracic anastomosis group than in the cervical anastomosis group (4 of 36 patients (11%) vs. 11 of 36 patients (31%); p = 0.040). The hospital stay was significantly shorter in the intrathoracic anastomosis group compared to the cervical anastomosis group (14 (range 10–110) vs. 26 days (range 12 – 105); p = 0.012). Wound infection and temporary paresis of the recurrent laryngeal nerve occurred significantly more often in the cervical anastomosis group compared to the intrathoracic anastomosis group (28% vs. 0%; p = 0.002 and 11% vs. 0%; p = 0.046). The overall In-hospital mortality rate was 6% (4 of 72 patients) without any differences between the study groups.

**Conclusions:**

The present data support the assumption that the transthoracic approach with an intrathoracic anastomosis compared to a cervical esophagogastrostomy is the safer and more beneficial procedure in patients with carcinoma of the lower and middle third of the esophagus due to a significant reduction of anastomotic leakage, wound infection, paresis of the recurrent laryngeal nerve and shorter hospital stay.

## Background

Up to date two techniques of resection of the esophagus are common [1,2]. In cases of cancer of the gastroesophageal junction some authors prefer the transhiatal approach with collar anastomosis in order to reduce early postoperative morbidity and mortality rates by avoiding thoracotomy and potential mediastinitis erosed by anastomotic leakage [3,4] despite an increased risk of leakage and anastomotic stricture formation [5]. Others favour the transthoracic resection to improve the cure rate by a more aggressive approach in which a more radical mediastinal lymphadenectomy can be achieved [6].

One major challenge after esophageal resection remains the occurrence of anastomotic leakage attended by mortality rates up to 50% [7]. The incidence of anastomotic leakage varied widely (3 to 50%) [1] independently of the placement of the anastomosis, which suggests that it is potentially a definitional problem. Some authors only count clinically significant leakages, whereas others also include subclinical, only radiographically detected leakages.

Although most study groups present their surgical outcome dependent on the surgical approach (either transthoracic or transhiatal) [1,2] we tried to investigate the influence of the position of the esophagogastrostomy (either cervical or intrathoracic) in a single center matched pair analysis.

## Patients and methods

The matched pair analysis was performed at the Department of General, Visceral and Transplantation Surgery, University Hospital of the RWTH Aachen, Germany. The study was conducted in accordance with the study protocol, the Declaration of Helsinki and applicable regulatory requirements. The study was approved by the ethics committee of the faculty of medicine of the RWTH University of Aachen. The approval number is EK 105/11.

Between June 2009 and June 2010 36 consecutive patients received an esophageal resection due to esophageal cancer in our department. All patients underwent *Ivor Lewis* subtotal esophagectomy with two-field lymph node dissection. In brief, all resections were performed by initial abdominal exploration through an upper midline laparotomy. The stomach was mobilized on the right gastric and gastroepiploic arteries. The left gastric artery was divided at its origin, and all lymph nodes along the celiac axis and its three branches along the left aspect of the portal vein, in front of the inferior vena cava, along the diaphragmatic pillars were resected. A pyloromyotomy was not performed routinely. With a right anterolateral thoracotomy, the chest was entered through the fifth intercostal space. The azygos vein arch was divided, and the esophagus was dissected from esophagogastric junction to the apex of the chest. Complete lymph node dissection of the dorsal mediastinum including subcarinal lymph nodes was performed. A resection of the thoracic duct was not performed routinely. Denudation of the lesser curvature was usually performed in the pleural cavity. After resection of the specimen, an end-to-side anastomosis was constructed between the esophagus and the stomach. The anastomosis was located in the apex of the chest and was delivered by a circular stapler device. None of the patients were excluded.

For each patient in the intrathoracic group, patients who received a cervical anastomosis in the time between January 2007 and May 2009 were selected from our database of patients (n = 60) in the same institution who matched the following criteria: age and tumor stage.

Among patients with cervical anastomosis in case of adenocarcinoma a transhiatal resection of the esophagus with left-sided cervical hand sutured end-to-end esophagogastric anastomosis was carried out, whereas in case of squamous cell carcinoma abdomino-right-sided-thoracic resection with two-field lymphadenectomy and left-sided cervical hand sutured end-to-end esophagogastric anastomosis was performed. Mobilisation of the gastric tube and lymph node dissection was equal to the technique described above. All patients with thoracotomy received a right-sided chest tube insertion.

Prospectively collected data from these patients were reviewed retrospectively. The observation period was the initial hospital stay. We analyzed the following clinical markers representing the early perioperative outcome: resected lymph nodes, operation time, intensive care stay (ICU), re-ICU-stay, overall hospital stay, In-hospital mortality, blood substitution intra- and postoperatively, postoperative bleeding, pneumonia, need of postoperative CPAP (non-invasive continuous positive airway pressure), re-intubation, anastomotic leakage, revision operation, wound infection, thrombemboli and chylothorax. In case of postoperative dysphony a larnygoscopy was performed to document potential laryngeal nerve palsy.

All patients were discussed at a weekly multidisciplinary meeting, and treatment strategies were developed and tailored for individual patients. In general, patients with squamous cell carcinoma were offered neoadjuvant radiochemotherapy in case of tumor stage II and III. Since 2007 patients with adenocarcinoma (stage II and III) received neoadjuvant chemotherapy according to the MAGIC Trial [8].

Preoperatively, all patients received a thoracic peridural catheter. After surgery, all patients returned to the intensive care unit and were extubated on the same day. All patients received a restrictive volume regime. All patients received a nasojejunal feeding tube during surgery which was removed on day 5 after surgery. Oral feeding started on day 5 after surgery. Before return to solid food a gastrografin swallowing (50 to 100 ml) examination was performed in all patients. In case of radiographically or clinically suspected anastomotic leakage endoscopy was performed. In cases of obvious leakage among patients with cervical anastomosis a nutrition tube was inserted into the duodenum endoscopically for enteral nutrition until spontaneous closure of the defect occurred. In cases of leakage of thoracic anastomosis a double-layered self-expandable stent was inserted with patient under sedation. Each stent was individually adapted in diameter and length dependent on the leakage size, and subjected to the distance between defect and upper esophageal sphincter. The location of the stent was visualized and controlled radiographically during the implantation.

### Statistical analysis

Means and standard deviations (notation: mean ± SD) as well as frequencies and percentage were given to describe the data. In case of not normal distribution data is represented as median and range. According to the matched pairs study design, we used paired t-tests to compare means between the intrathoracic and cervical anastomosis groups, e.g. the mean ICU stay. Further the *Cochrane Mantel Haenszel* test stratified by pairs was used to compare proportions between the intrathoracic and cervical anastomosis groups, e.g. proportion of anastomotic leakage. Statistically significance was assessed if the *p*-value of the corresponding test fell below the significance level of 0.05. Due to the small sample size our statistical analysis was limited to bivariate statistical analysis. Thus no stratified (multivariate) analysis could be conducted because of lack of model fit. We used SAS® V9 under Windows XP for computations.

## Results

Patients’ preoperative characteristics including risk factors presented in Table 1 did not show any significant differences between the two groups except the fact that in the intrathoracic group significantly more patients received preoperative chemotherapy than in the cervical group (p = 0.003). 18 ± 8 lymph nodes were resected in the intrathoracic group whereas 16 ± 7 lymph nodes were resected in the cervical group (p = 0.539).

**Table 1:** Patients characteristics risk factors in dependency of type of anastomosis

	**Intrathoracic (n = 36)**	**Cervical (n = 36)**	**p-value**
Age	62 ± 12	62 ± 10	
Gender			
- male	34 (94%)	30 (83%)	0.157
- female	2 (6%)	6 (17%)	
ASA-classification			
−2	18 (50%)	19 (53%)	
−3	18 (50%)	17 (47%)	0.819
Cardiac disease	9 (25%)	9 (25%)	1.000
Pulmonary disease	8 (22%)	10 (28%)	0.527
Hypertension	24 (67%)	19 (53%)	0.225
Diabetes	10 (28%)	8 (22%)	0.593
Abuse of nicotine	17 (47%)	17 (47%)	1.000
Abuse of alcohol	13 (36%)	13 (36%)	1.000
Histology			
-adenocarcinoma	29 (81%)	26 (72%)	0.317
-squamous cell carcinoma	7 (19%)	10 (28%)	
UICC stage			
- I	16 (44%)	15 (42%)	
-II	8 (22%)	9 (25%)	
-III	10 (28%)	11 (31%)	
-IV	2 (6%)	1 (3%)	
Grading			
2	15 (42%)	16 (44%)	0.796
3	21 (58%)	20 (56%)	
Radiatio preoperative	6 (17%)	4 (11%)	0.414
Chemotherapy preoperative	20 (56%)	7 (19%)	**0.003**

Neither operation time (261 ± 53 min vs. 238 ± 60 min; p = 0.087) nor ICU stay (3.4 ± 6.7 days vs. 5.3 ± 6.9 days; p = 0.232) revealed any statistical differences in-between the intrathoracic and the cervical group. The median hospital stay was significantly lower in the intrathoracic group in comparison to the cervical group (14 (10 – 110) vs. 26 (12 – 105); p = 0.012).

Complication rates were widely similar between the groups (Table 2). Anastomotic leakage rate was significantly lower in the intrathoracic group than in the cervical group (11% vs. 31%; p = 0.040). Wound infection and temporary paresis of recurrent laryngeal nerve occurred significantly more often in the cervical group than in the intrathoracic group (wound infection: 28% vs. 0%; p = 0.002; temporary paresis of recurrent laryngeal nerve: 11% vs. 0%; p = 0.046). In 50% off all cases the recurrent nerve paresis went to normal function within 12 weeks. There was no significant difference regarding blood substitution intraoperatively (17% intrathoracic vs. 8% cervical; p = 0.317) and postoperatively (22% intrathoracic vs. 33% cervical; p = 0.248) comparing study groups.

**Table 2:** Operation details and complications in dependency of type of anastomosis

	**Intrathoracic (n = 36)**	**Cervical (n = 36)**	**p-value**
Operation time in min	261 ± 53	238 ± 60	0.087
Thoracotomy	36 (100%)	7 (19%)	**<0.001**
ICU stay in days	3.4 ± 6.7	5.3 ± 6.9	0.232
Re ICU	7 (19%)	6 (17%)	0.739
Median Hospital stay in days	14 (10 – 110)	26 (12 – 105)	**0.012**
In-hospital mortality	1 (3%)	3 (8%)	0.310
Blood transfusion intraoperatively	6 (17%)	3 (8%)	0.317
Blood transfusion postoperatively	8 (22%)	12 (33%)	0.248
Patients with lymph nodes positive	17 (47%)	18 (50%)	0.655
Resected lymph nodes	18 ± 8	16 ± 7	0.539
Bleeding	1 (3%)	0 (0%)	0.317
Pneumonia	7 (19%)	7 (19%)	1.000
CPAP	10 (28%)	10 (28%)	1.000
Re-intubation	9 (25%)	9 (25%)	1.000
Anastomotic leakage	4 (11%)	11 (31%)	**0.040**
Revision operation	3 (8%)	2 (6%)	0.655
Wound infection	0 (0%)	10 (28%)	**0.002**
Thrombemboli	4 (11%)	3 (8%)	0.706
Paresis of recurrent laryngeal nerve	0 (0%)	4 (11%)	**0.046**
Chylothorax	1 (3%)	1 (3%)	1.000
Radiatio postoperative	3 (8%)	3 (8%)	1.000
Chemotherapy postoperative	17 (47%)	10 (28%)	0.108

## Discussion and conclusions

The present investigation is based on a total amount of 72 patients with esophageal cancer. Thirty six consecutive patients who had undergone surgery for carcinoma of the esophagus by an intrathoracic anastomosis, and 36 patients fulfilling matching criteria as stated above who received esophageal resection including a cervical anastomosis. Aim of this study was not only to compare the operative procedures in a single center study but also to serve as a quality assurance of our department since therapeutic strategy was changed in 2009.

Matched pairs analysis is to be preferred in our setting, because the comparison with an historical control group is likely to be biased by heterogeneity between groups. The matched pairs are similar in respect to the most important factors which may bias treatment differences. Analyzing patients’ preoperative risk factors and characteristics no significant differences in-between study groups were found supporting the fact that matching criteria were met. The intrathoracic group received significantly more often chemotherapy pre- and postoperatively which is due to the fact that since 2007 patients with adenocarcinoma (stage II and III) received neoadjuvant chemotherapy according to *Cunningham* et al. [8]. However, although the intrathoracic group received preoperative chemotherapy significantly more often, the operative complications rates were not higher than in the cervical group without neoadjuvant treatment.

Whether the anastomosis should be performed cervically, allowing wider margins of resection and elevated risk of paresis of recurrent laryngeal nerve, or whether intrathoracic anastomosis is favoured, with decreased resection margins is still a debatable issue in reconstruction after esophagectomy [9]. It is discussed controversially whether intrathoracic anastomosis reduces anastomotic leakage or not. While in many studies the occurrence of anastomotic leakage was lower when applying an intrathoracic anastomosis [1,10-14], others did not find any differences between the two surgical procedures [9,15,16]. In our series anastomotic leakage occurred in 21% of all patients favouring intrathoracic anastomosis in comparison to cervical anastomosis (11% vs. 31%). A possible explanation might be that the intrathoracic placement of the anastomosis offers a better blood supply of the gastric interposition. Since all intrathoracic anastomosis were stapled and all cervical anastomosis were hand-sewn the technique of anastomosis might have an influence on leakage rates, although in other studies no influence of the suture method on the leakage rate was found [17-19]. In a systematic review of eight randomized clinical trials no difference was found comparing hand-sewn versus stapled techniques [20]. However, the incidence of anastomotic leakage following esophageal surgery varies widely (3% to 50%) also depending on the definition of leakage [1].

According to many studies intrathoracic anastomosis which is always associated with thoracotomy is accompanied by a higher risk for perioperative complications, especially of pulmonary kind and higher mortality rates [3,4,21]. In contrast to the findings we found widely similar perioperative complication rates in our study groups. Neither the presence of pulmonary complications nor the need of re-intubation was increased in the intrathoracic group. One possible reason might be that 19% of the patients of the cervical group received a thoracotomy as well. Wound infection was significantly elevated in the cervical group since drainage of anastomotic leakage in case of cervical anastomosis is achieved through the cervical wound. The In-hospital mortality rate was slightly higher in the cervical anastomosis group without reaching significant difference, which is in accordance to *Rindani et al. and Chasseray et al.* who found similar mortality rates for transthoracic and transhiatal resections [22,23].

In our series the mean lymph node harvests following each procedure were comparable with those reported by other centres, which ranged from 9 to 31 [4,24]. Some authors mentioned that a disadvantage of the transhiatal resection is a reduced transthoracic lymph node dissection, which is unfortunately limited to the lower posterior, mediastinal lymph nodes [25,26]. However, in accordance to *Morgan* et al. we found a similar amount of resected lymph nodes in both study groups [24].

High median hospital stay might be due to the fact that patients with anastomotic leakage stayed significantly longer than patients without anastomotic leakage which is comparable to the findings of previous studies [27]. Despite the assumption that a transthoracic approach with an intrathoracic anastomosis is considered to be a more invasive procedure than a transhiatal approach with cervical anastomosis, we neither found a prolonged ICU nor hospital stay. Although there might be more reasons other than placement of the anastomosis (e.g. introduction of fast track regime), the hospital stay was significantly lower since the new therapeutical strategy was established, indicating that the placement of an intrathoracic anastomosis represents a save and feasible routine procedure without higher perioperative complication rates in comparison to the placement of a cervical anastomosis. However, *Kayani* et al. showed that there is currently insufficient evidence to show a significant difference between cervical and intrathoracic anastomosis with respect to post-operative complications and hospital mortality [28].

In conclusion of our study, after subtotal esophagectomy the intrathoracic anastomosis compared to the collar anastomosis seems to be slightly favourable in terms of perioperative outcome. Due to the change of the surgical approach we were able to reduce anastomotic leakage rates but not to improve the extent of mediastinal nodal resection. No conclusions can be drawn from this data concerning the long-term survival or long-term complications like anastomotic stenosis.

## Competing interests

The author(s) declare that they have no competing interests.

## Authors' contributions

Christian D. Klink is the responsible main author of this article. He is responsible for its content. Marcel Binnebösel and Jens Otto have made substantial contributions to conception and design of this article. Gabriele Boehm has made substantial contributions to acquisition of data, analysis and interpretation of data. Klaus T. von Trotha and Joachim Conze have been involved in drafting the manuscript and revising it critically for important intellectual content. Ralf-Dieter Hilgers is responsible for the statistical analysis of this article. Ulf P. Neumann and Marc Jansen have given final approval of the version to be published. All authors read and approved the final manuscript.
